# The impact of locus coeruleus degeneration on pair bonding and other Alzheimer’s disease‐relevant phenotypes in monogamous prairie voles

**DOI:** 10.1002/alz.094959

**Published:** 2025-01-09

**Authors:** Michael A. Kelberman, Zoe R Donaldson, Kelly Winther, Emily Franks, David Weinshenker

**Affiliations:** ^1^ University of Colorado Boulder, Boulder, CO USA; ^2^ Emory University, Atlanta, GA USA

## Abstract

**Background:**

Alzheimer’s disease (AD) fractures social relationships, extending the burden of disease beyond the affected individual and onto caregivers and loved ones. One brain region known to be crucial in regulating social behaviors is the noradrenergic locus coeruleus (LC), a region that becomes dysfunctional as AD progresses. Unfortunately, deficits in social bonding have been relatively understudied in AD, due in part to the limited social behavioral repertoires of mouse and rat models of AD. Here, we used monogamous prairie voles, which display many human‐relevant complex social behaviors, to assess the impact of LC degeneration on social bonding and other AD‐relevant phenotypes.

**Methods:**

Prairie voles (male and female, 3 – 6 months of age) were administered the selective LC neurotoxin DSP4 or saline (i.p., administered twice separated by a week). Voles were paired a week after their last injection and tested on partner preference tests 1, 3, and 7 days post‐pairing, timepoints designed to test for the presence and duration of bond impairment. Animals were then tested on sleep latency (arousal), Y‐maze (working memory), novelty‐suppressed feeding (anxiety‐like phenotypes), and resident intruder (social behavior) tasks to assess other AD‐relevant phenotypes. Vole pairs were then separated and singly housed in separate vivariums. After 4 weeks of isolation, they were re‐tested in the same battery of behavioral tests and in a final partner preference.

**Results:**

DSP4 administration impaired bond formation in male but not female voles, even after 7 days of cohabitation, a striking result as other manipulations that impair bond formation typically delay rather preclude bonding. Further supporting a lack of pair bonding, DSP4 treated males displayed lower levels of aggression in a resident intruder task while still co‐housed with their partner. Meanwhile, partner separation increased anxiety‐like behavior in DSP4‐treated males, suggesting an interaction with a well‐delineated social stressor. Female behavior was unaffected by DSP4 treatment. Confirmation of LC lesions with HPLC and IHC, and behavioral analysis of partner preference after separation are ongoing.

**Conclusions:**

This study shows that LC degeneration may contribute to social behavior abnormalities observed in prodromal AD. It further suggests that LC degeneration may exaggerate behavioral deficits following the loss of a partner.

